# A Clear Mystery: Recognizing Lipomatosis of the Nerve

**DOI:** 10.7759/cureus.5290

**Published:** 2019-07-31

**Authors:** Khaled M Gaber, Tomas Marek, Jürgen Meixensberger, Robert Spinner, Mark A Mahan

**Affiliations:** 1 Department of Neurosurgery, University Hospital Leipzig, Leipzig, DEU; 2 Department of Neurosurgery, Mayo Clinic and Foundation, Rochester, USA; 3 Department of Neurosurgery, University of Utah, Salt Lake City, USA

**Keywords:** brachial plexus, fibrolipomatous hamartoma, lipofibromatous hamartoma, overgrowth, lipomatosis of the nerve

## Abstract

Lipomatosis of the nerve (LN) commonly presents with neurologic dysfunction due to massive fibro-fatty enlargement of the peripheral nerves. It is uniquely associated with adipose proliferation in the subcutaneous tissue and muscle in the innervated territory, along with osseous abnormalities. Herein, we present the case of a 56-year-old woman who presented with severe right ulnar distribution pain involving the medial forearm and hand (9/10 on a numerical rating scale), declining right-hand strength, movement-dependent hypoesthesias, paresthesias, and a pronounced claw deformity of the right hand with intrinsic atrophy. Electrodiagnostic studies demonstrated pronounced fibrillations, decreased voluntary activation, and minimal collateral reinnervation in the abductor digiti minimi and abductor pollicis brevis, consistent with dysfunction of the lower trunk of the right brachial plexus. Magnetic resonance imaging (MRI) and computed tomography (CT) of the brachial plexus were interpreted as a tumor on the right supra- and infraclavicular brachial plexus. At surgery, the brachial plexus was embedded in relatively tight connective tissue with a typical lipoma posteriorly. The lipoma was resected, and the plexus was explored extensively. This case is the 10th report of LN involving the brachial plexus and demonstrated the cardinal features of LN. It provides insight into the pattern of lesions associated with innervation by LN.

## Introduction

Lipomatosis of the nerve (LN) is an uncommon disorder of the peripheral nerves characterized by interfascicular fibro-fatty proliferation within the epineurium that leads to massive nerve hypertrophy and consequent symptoms secondary to mass effect. A unique feature associated with LN is nerve territory overgrowth distal to the lesion, which occurs in 62% of cases [1] and can affect bony and soft-tissue structures. The soft-tissue overgrowth includes extraneural lipomas [2] and intramuscular fatty infiltration within innervated muscles. Although it is a benign lesion, the distal overgrowth is commonly progressive [3].

The fibro-fatty proliferation within the epineurium provides the LN a pathognomonic appearance on magnetic resonance imaging (MRI) with coaxial cables on axial images and a spaghetti-like appearance on longitudinal planes [3-4]. Despite this pathologic consistency, the condition is frequently diagnosed inaccurately, leading to the use of outdated terms like fibrolipomatous hamartoma, lipofibromatous hamartoma, macrodystrophia lipomatosa, neural lipofibroma, and fibrolipoma [1].

We report a case of LN affecting the brachial plexus that was not recognized before surgery. This case was presented at the 2019 World Federation of Neurosurgical Societies Theoretical & Practical Course of Peripheral Nerve & Brachial Plexus, Frankfurt, Germany, Sept. 24-26, 2018, for unusual features and the uncertain diagnosis (Gaber K: Lipomatous hyperplasia of the brachial plexus). In retrospect, all of the canonical features of LN were present. The pattern of pathology, in this case, was also unique and demonstrates the particular irregularity of LN in its manifestations.

## Case presentation

A 56-year-old woman presented complaining of severe right arm pain of approximately 18 months duration. She was having ulnar-distribution pain involving the medial forearm and extending into the hand (9/10 on a numerical rating scale). She noted declining strength of the right hand and movement-dependent hypoesthesias and paresthesias. She was otherwise healthy and without a family history of hereditary tumor syndromes.

A clinical examination revealed normal wrist and finger extension at Motor Research Council grade 5/5, wrist flexion 5/5, finger abduction 3/5, and thumb abduction and adduction 0/5. There was a pronounced claw deformity of the right hand with intrinsic atrophy.

**Figure 1:**
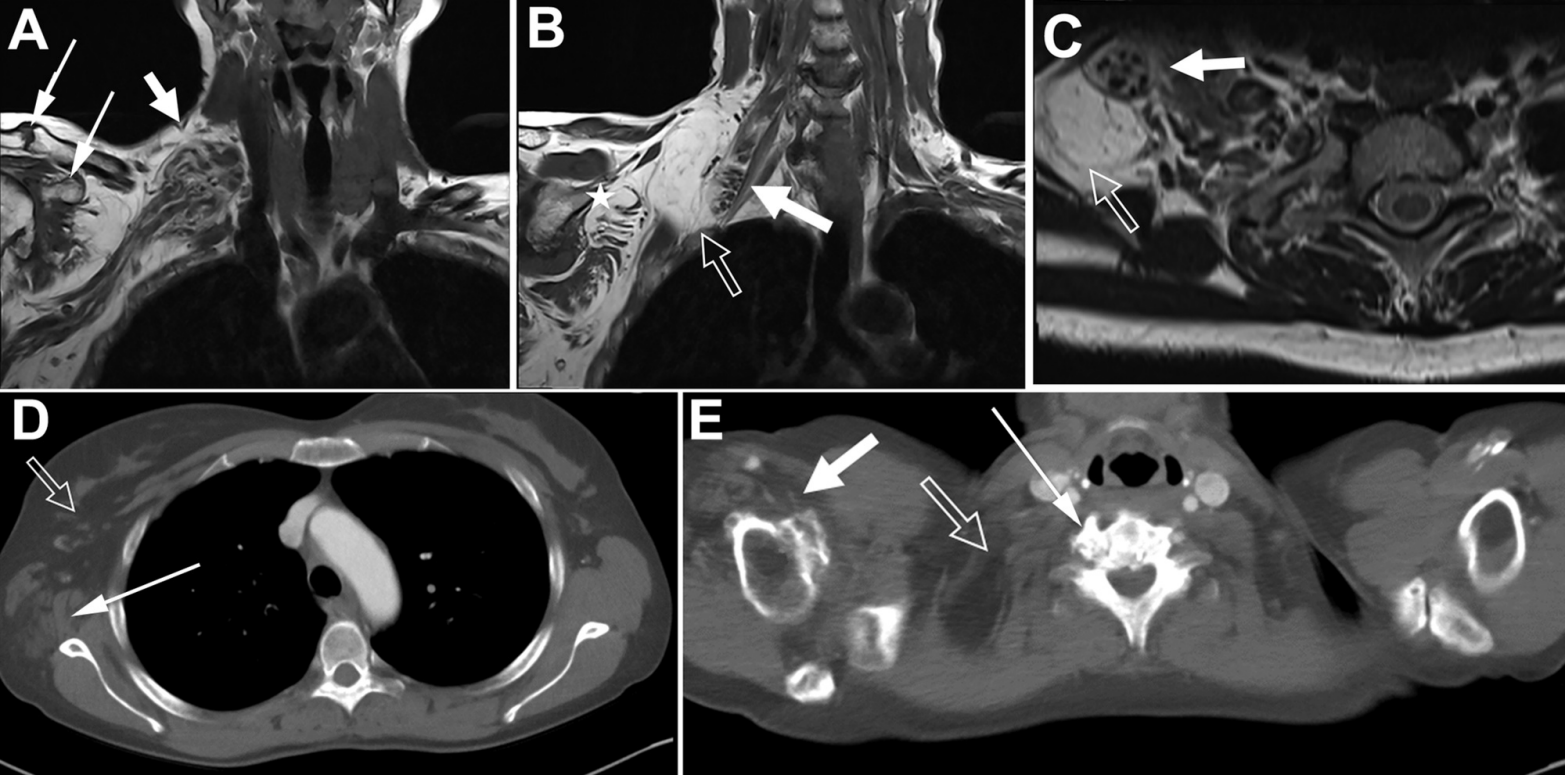
MRI and CT images of the right brachial plexus before exploration. (A) The T2-weighted coronal image demonstrates the enlargement of the entire brachial plexus (thick arrow), as well as overgrowth of the acromion and coracoid process (thin arrows); (B) An extraneural lipoma (hollow arrow) was posterior to the affected nerves (solid arrow) after they exited the scalene triangle. The nerves of the brachial plexus demonstrate the classic cable-like LN appearance involving the upper, middle, and lower trunks. Fatty infiltration of the subscapularis muscle is visible (star); (C) The same relationship can be seen in the axial T2-weighted cross-sectional imaging depicting the cable-like appearance of the brachial plexus (solid arrow) and the extraneural lipoma (hollow arrow); D & E: Computed tomography images of the chest before brachial plexus exploration; (D) Axial image at the arch of the aorta demonstrates asymmetric subcutaneous adipose (hollow arrow) and fatty infiltration of the latissimus dorsi muscle (thin arrow); (E) Axial image at the level of C7-T1 demonstrates marked lateral neuroforaminal osteophyte formation (thin arrow), bulky hypertrophy of the brachial plexus with associated lipoma (hollow arrow), and osteophyte formation of the right humerus (solid arrow) in sharp comparison to the normal left side.

An MRI of the cervical spine revealed foraminal stenosis at C6-7 and C7-T1 (not shown). Because of the pain in her arm, cervical decompression was recommended, and she underwent an anterior cervical discectomy. Postoperatively, her symptoms did not improve after discectomy, and she instead noted worsening pain. Electrodiagnostic studies demonstrated pronounced fibrillations, decreased voluntary activation, and minimal collateral reinnervation in the abductor digiti minimi and abductor pollicis brevis, consistent with injury to the lower trunk of the right brachial plexus. MRI (Figure 1A-C) and computed tomography (CT) (Figure 1D-E) scans of the right brachial plexus were interpreted as a tumor on the right supra- and infraclavicular brachial plexus that was weakly contrast-enhancing.

**Figure 2:**
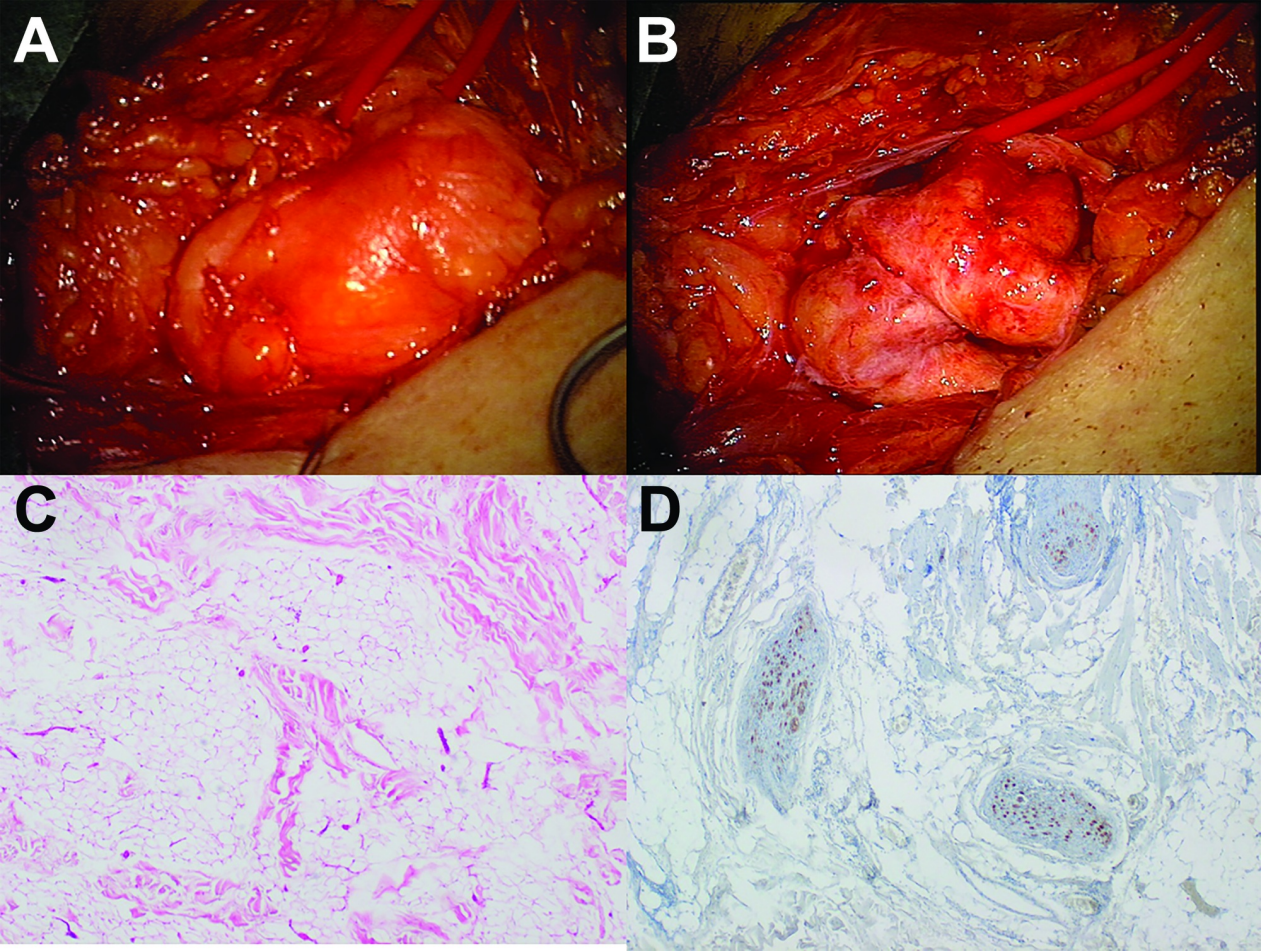
Intraoperative photographs and histological imaging A & B: Intraoperative photographs demonstrate (A) the extraneural, encapsulated lipoma and (B) the massively enlarged, redundant, and fibrotic elements of the brachial plexus. C & D: Photomicrographs of histopathologic sections depict mature, uniform adipocytes without atypical nucleus separated by dense fiber-rich connective tissue (C: hematoxylin and eosin, x40). Small nerve fibers were found to penetrate the connective tissue septa of the lipoma (D: S100, x40).

Surgical exploration for neurolysis and removal of the associated lipoma was recommended. Intraoperatively, the brachial plexus was embedded in relatively tight connective tissue with a typical lipoma posteriorly. The lipoma was resected, and the plexus was explored extensively, resulting in the removal of the tight connective tissue (Figure 2A-B). At the conclusion of the case, the nerves were slack and widely decompressed but of notably firm consistency. Pathologic specimens showed a mass of mature, uniform adipocytes separated by dense fibrous septa (Figure 2C), compatible with lipoma. Small nerve fibers were identified in the fibrous septa with antibody staining to S100 (Figure 2D).

Immediately postoperatively, the patient reported decreasing pain and hypoesthesia. She also noted subjective improvement of the right-hand agility. Unfortunately, she had neurologic worsening a few months after surgery and declined further treatment and imaging.

Reinterpretation

Reevaluation of the MRI of the right brachial plexus demonstrated pathognomonic spaghetti-like fascicular enlargement and fatty displacement on the coronal plane and coaxial cables on the sagittal plane (Figure 1A-C). The lipofibromatous involvement extended from the C5-T1 spinal nerves to the cord level, with the largest proliferation affecting the upper trunk. Imaging did not cover the whole affected extremity so the full distal extent of the LN is not known. Nerve territory overgrowth affected soft tissues and bone. Soft-tissue overgrowth included the extraneural lipoma contiguous to the brachial plexus, a fatty muscle infiltration in various muscles in the latissimus dorsi, subscapularis, and teres major, and the enlargement of the subcutaneous fat around the latissimus dorsi (Figure 1D). The cervical spine imaging demonstrated asymmetric hypertrophic bony changes of the neural foramen and a circumferential osseous canal of the vertebral artery (Figure 1E), which is atypical of fusion changes and predated the fusion by history. Other bony abnormalities noted included exostoses of the glenohumeral joint and overgrowth of the coracoid, acromion, and scapula. Although the patient’s hand clearly demonstrated atrophy and not distal hypertrophy, it is uncertain whether other muscles of the upper arm or forearm were involved.

## Discussion

This case presents a fascinating depiction of LN involving the brachial plexus and its spectrum of associated abnormalities caused by nerve territory overgrowth and neuropathy. We have systematically reviewed 2,465 publications related to LN and have identified 1,025 cases of confirmed or probable LN, based on the radiographic and pathologic diagnosis [1]. In that extensive review of the world’s literature on LN, there were only nine cases of LN involving the brachial plexus. This report adds the 10th reported case of LN affecting the brachial plexus. Eight of the nine reported cases of LN of the brachial plexus in the literature had associated nerve territory overgrowth [6]. An extraneural lipoma(s) is an exemplary manifestation of LN, present in 7% of cases identified in a systematic review of the literature on LN. While the entirety of the brachial plexus appears affected by LN, not all muscles of the imaged shoulder region were affected by adipose infiltration; selective adipose replacement was present in the latissimus dorsi, teres major, and subscapularis muscles (consistent with the involvement of the proximal branches of the posterior cord). LN appears to manifest with osseous hypertrophy at the joints [5], often with exquisite exostoses and osteochondromas. Lastly, the consequences of LN are not always hypertrophy. The patient had muscle atrophy from denervation, which produced a Gilliatt-Sumner hand consistent with lower trunk plexopathy. Previously, LN-associated neuropathy has been assumed to be due to entrapment, but neuropathy intrinsic to the disease cannot be excluded.

The missed diagnosis of LN before surgical exploration is unlikely to have changed the outcome. The profound atrophy of the hand intrinsic muscles from a lower trunk plexopathy is often irreversible. Surgical decompression is recommended for LN lesions when neuropathy arises [7]; in most cases, this involves a carpal tunnel release. In this case, surgical decompression was achieved with lipoma resection. Other procedures that might be beneficial include other soft-tissue debulking and osteotomy. In carefully selected cases, amputation of the overgrown structure(s) might be considered if it would improve function. In this case, a scalenectomy and/or first thoracic rib resection could have been considered for further decompression; however, this might not have improved the outcome and would, therefore, present unwarranted risks. A biopsy was not necessary for diagnosis, as the MRI is pathognomonic for LN. LN of the brachial plexus is particularly rare, and there was a reasonable concern for alternative pathology given possible contrast enhancement.

## Conclusions

The appearance of LN on MRI is pathognomonic, and the diagnosis needs to be considered, even at an unusual site like the brachial plexus. This LN case was associated with evidence of soft-tissue and bony overgrowth; however, there was no evidence of distal limb overgrowth. Cases like this one show that there is much more to be learned about LN and the ramifications to anatomy under its innervation.

## References

[1] Marek T, Spinner RJ, Syal A, Mahan MA (2019). Strengthening the association of lipomatosis of nerve and nerve-territory overgrowth: a systematic review. J Neurosurg.

[2] Marek T, Amrami KK, Mahan MA, Spinner RJ (2018). Intraneural lipomas: institutional and literature review. Acta Neurochir (Wien).

[3] Mahan MA, Niederhauser BD, Amrami KK, Spinner RJ (2014). Long-term progression of lipomatosis of nerve. World Neurosurg.

[4] Marom EM, Helms CA (1999). Fibrolipomatous hamartoma: pathognomonic on MR imaging. Skeletal Radiol.

[5] Mahan MA, Amrami KK, Spinner RJ (2013). Sciatic nerve lipomatosis and knee osteochondroma. J Neurosurg.

[6] Marek T, Amrami KK, Spinner RJ (2019). Lipomatosis of the brachial plexus with associated overgrowth and macrodactyly. Clin Anat.

[7] Marek T, Spinner RJ, Syal A, Wahood W, Mahan MA (2019). Surgical treatment of lipomatosis of nerve: a systematic review. World Neurosurg.

